# Long-term outcomes of nasopharyngeal carcinoma patients with T1-2 stage in intensity-modulated radiotherapy era

**DOI:** 10.7150/ijms.68394

**Published:** 2022-01-01

**Authors:** Xiaoshuang Niu, Fen Xue, Peiyao Liu, Chaosu Hu, Xiayun He

**Affiliations:** 1 Department of Radiation Oncology, Fudan University Shanghai Cancer Center, Shanghai 200032, China; 2 Department of Oncology, Shanghai Medical College, Fudan University, Shanghai 200032, China; 3 Shanghai Key Laboratory of Radiation Oncology, Shanghai 200032, China

**Keywords:** nasopharyngeal carcinoma, T1-2, long-term outcomes, intensity-modulated radiotherapy

## Abstract

**Objectives:** To evaluate long-term outcomes and late toxicities of nasopharyngeal carcinoma (NPC) patients with T1-2N0-3M0 stage in intensity-modulated radiotherapy (IMRT) era.

**Materials and Methods:** From June 2005 to October 2013, 276 patients confirmed T1-2N0-3M0 NPC treated with IMRT were reviewed, with 143 (51.8%) N0-1 disease and 133 (48.2%) N2‐3 disease. Among them, 76.4% received chemotherapy. The prescribed doses given to the primary tumor and lymph nodes were 66Gy in 30 fractions.

**Results:** After a median follow-up of 103 months, the 5-year and 10-year overall survival (OS) were 90.6% and 79.2%. The 5-year and 10-year local control (LC) rate, regional control (RC) rate and distant metastasis free survival (DMFS) were 97.0% and 91.9%, 94.1% and 92.2%, 89.4% and 87.0%, respectively. The 5-year and 10-year OS, RC rate and DMFS of N0-1 compared with those of N2-3 were 98.6% vs. 82.0% and 86.8% vs. 70.9% (P=0.000), 99.3% vs. 88.3% and 99.3% vs. 84.1% (P=0.000), 97.9% vs. 80.1% and 95.7% vs. 77.5% (P=0.000). The incidence of 3-4 late toxicities were low and mainly xerostomia and hearing deficit. The rates of radiation-induced cranial nerve palsy and temporal necrosis were 2.5% and 2.5%, respectively. Eighteen patients had the second primary tumor, of whom eight were lung cancer, six were head and neck cancer, four were others.

**Conclusions:** Satisfactory locoregional control was achieved in T1‐2N0-3M0 NPC treated with IMRT. Distant metastasis was the main failure cause and N2-3 was the main adverse prognostic factor. Second primary tumor occurred 6.5% and negatively impacted OS in NPC.

## Introduction

Radiotherapy (RT) is the main definitive therapy for non-metastatic nasopharyngeal carcinoma (NPC) due to its relative high radiosensitivity and anatomic constraints. The 5-year survival rate for stage I-IV NPC after 2-dimensional radiotherapy (2DRT) was 59-75% 1-3. New radiotherapy technique, intensity-modulated radiotherapy (IMRT) has improved dose delivery to NPC and reduced dose to normal tissues, the 5-year survival rate reached 80-86% with lower toxicities 4-6. Since little research was reported on long-term outcomes of NPC patients 7-9 and different subgroups have their own characteristics 10-12, a retrospective analysis of 276 patients with T1-2N0-3 NPC has been conducted. Moreover, this study aimed to analyze the long-term outcome and late toxicities to further develop the stratification of T1-2N0-3 disease.

## Materials and methods

### Patients

From June 2005 to October 2013, 276 histologically diagnosed non-metastatic NPC patients with the 8th AJCC/UICC staging criteria T1-2N0-3M0 were enrolled in this study. All patients provided informed written consent before treatment. Initial assessment consisted of medical history and physical examination, blood routine and biochemistry tests, nasopharyngoscopy, enhanced magnetic resonance imaging (MRI) of the nasopharynx and enhanced MRI/CT of the neck. Other assessment included positron emission tomography-CT (PET-CT), or replaced by chest CT, abdominal ultrasound/CT and bone emission CT (stage N2-3). All patients were restaged according to the 8th AJCC/UICC staging criteria.

### Radiotherapy

All patients were treated with IMRT. The gross tumor volume (GTV) included primary nasopharyngeal tumor and positive lymph nodes. The clinical target volume (CTV) included the nasopharynx, parapharyngeal space, retropharyngeal lymph node, posterior one‐third of the nasal cavity and maxillary sinus, anterior clivus, pterygoid plates, inferior sphenoid sinus, and drainage of the neck (levels II, III, and Va in patients with N0 stage and levels II‐Vb in patients with N1‐3 stage). The prescribed dose given to primary tumor and the lymph nodes were 66Gy in 30 fractions (PTV-NX: GTV-NX +3-5 mm; PTV-LN: GTV-LN +3-5 mm). The PTV-60 covering the high-risk CTV and a 5-mm margin was prescribed 60Gy/30F. The PTV-54 covering the low-risk CTV and a 5-mm margin was prescribed 54Gy/30F. Radiotherapy was given once daily, 5 fractions per week.

### Chemotherapy

All N0 stage patients didn't receive chemotherapy. Most N1 stage (the diameter of lymph node ≥3 cm) and N2-3 stage patients received platinum‐based chemotherapy except intolerable. A total of 211 (76.4%) patients received chemotherapy, including induction chemotherapy (IC) ± concurrent chemotherapy (CCRT) or adjuvant chemotherapy (AC). CCRT was cisplatin 30-40 mg/m2 weekly during IMRT. Generally, the IC/AC regimens were delivered: TPF (docetaxel 60 mg/m2 d1+DDP 25 mg/m2 d1-3+5-FU 500 mg/m2 /d with 120-h infusion), PF (DDP 25 mg/m2 d1-3+5-FU 500 mg/m2 /d with 120-h infusion) or GP (gemcitabine 1.0g/m2 d1, d8+DDP 25mg/m2 d1-3). IMRT stated at 21 days after IC and AC stated at 28 days after the end of RT.

### Assessment and follow-up

Radiotherapy-related toxicities were graded by to the Radiation Therapy Oncology Group (RTOG). It was assessed every week during the radiotherapy. After treatment completion, follow-ups occurred every 3 months for the first 2 years, every 6 months from the third through the fifth year and annually thereafter. Routine follow-up included medical history, nasopharyngoscopy and physical examination. Enhanced MRI of the nasopharynx was performed every 6 to 12 months. Chest CT and ultrasonography of the abdomen were conducted once yearly. Bone emission CT was performed when there were clinical indications.

### Statistical analysis

SPSS 23.0 (SPSS Inc, Chicago, IL, USA) was used for statistical analysis in this study. Overall survival (OS) was calculated from the date of initiation of treatment to the date of death or last follow‐up. Local control (LC) was calculated from the date of initiation of treatment to the date of local failure or last follow‐up. Regional control (RC) was calculated from the date of initiation of treatment to the date of regional failure or last follow‐up. Distance metastasis‐free survival (DMFS) was calculated from the date of initiation of treatment to the date of metastasis or last follow‐up. Factors (P<0.2) were included in a multifactor Cox model to determine the independent prognostic factors. The duration of survival was measured from the time of treatment until death or the date of the last follow up visit for patients alive. A 2-sided P<0.05 was considered statistically significant.

## Results

### Patient characteristics and survival

**Figure 1:**
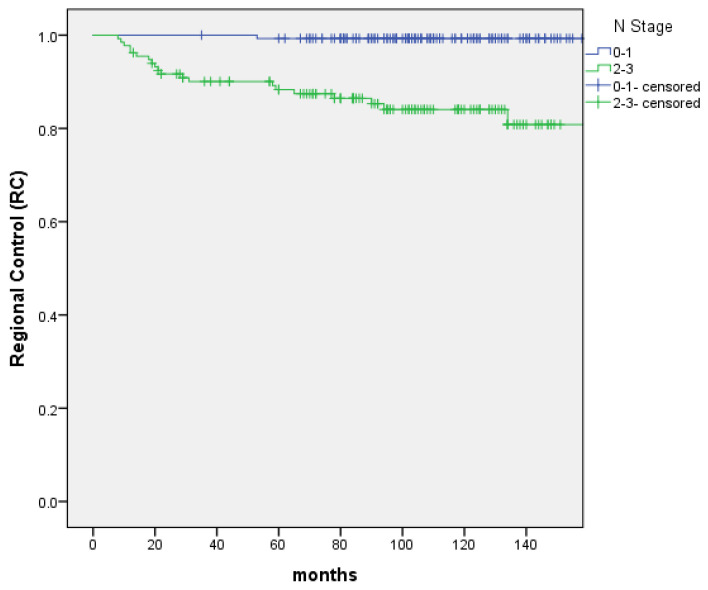
The RC rate between patients with N0-1 and N2-3 stage, respectively

**Figure 2:**
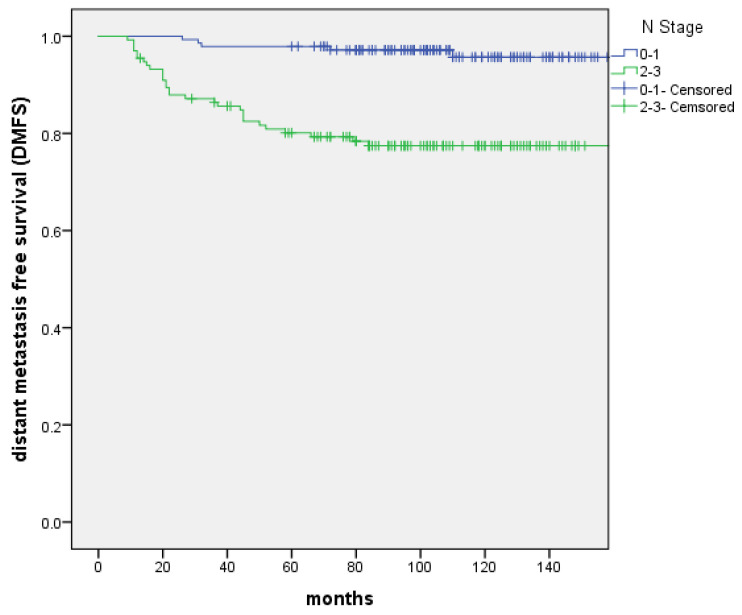
The distant metastasis-free survival (DMFS) between patients with N0-1 and N2-3 stage, respectively

**Figure 3:**
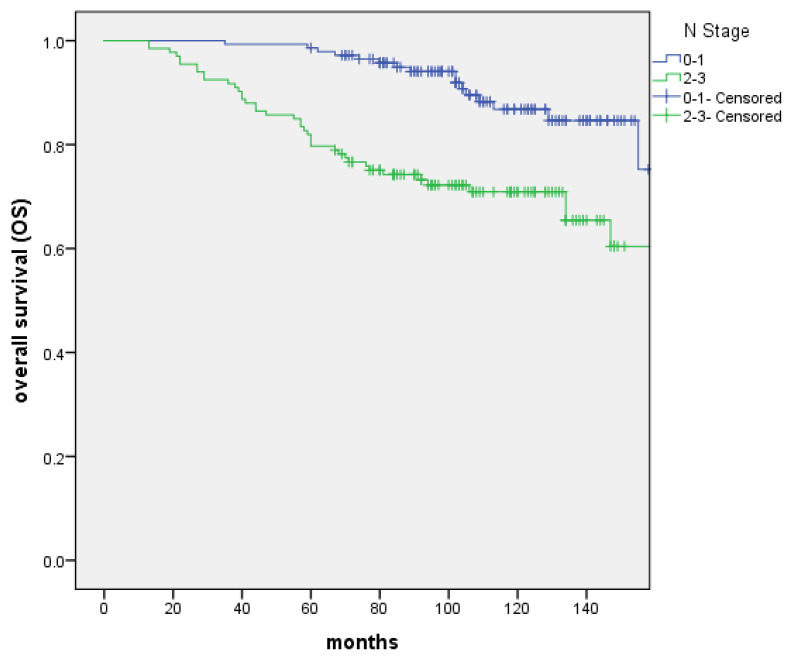
The overall survival (OS) between patients with N0-1 and N2-3 stage, respectively

From June 2005 to October 2013, 276 patients confirmed T1-2N0-3M0 NPC treated with IMRT were reviewed. The characteristics of patients were shown in Table 1. PET-CT was included in the analysis because it was more sensitive for the detection of distant metastases. The presence of distant metastases might be underdiagnosed without an initial PET-CT. The median follow-up was 103 months (range: 13 to 183 months). 17 patients were recurrence in nasopharynx. The 5-year and 10-year LC rate were 97.0% and 91.9%, and significant difference was not discovered between the T1 and T2 groups (P=0.505). 21 patients were recurrence in regional lymph nodes. The 5-year and 10-year RC rate were 94.1% and 92.2%, respectively. Patients with N0‐1 stage showed improved 5‐year and 10-year RC rate compared with N2-3 patients (99.3% vs. 88.3% and 99.3% vs. 84.1% (P=0.000); Figure 1). 34 patients were distant metastasis. The 5-year and 10-year DMFS were 89.4% and 87%, respectively. Patients with N0‐1 stage showed improved 5‐year and 10-year DMFS compared with N2-3 patients 97.9% vs. 80.1% and 95.7% vs. 77.5% (P=0.000); Figure 2). A total of 56 patients died. The 5-year and 10-year OS were 90.6% and 79.2%, and no significant difference was found between the T1 and T2 stage (P=0.171). The 5-year and 10-year OS of N0-1 compared with those of N2-3 were 98.6% vs. 82% and 86.8% vs. 70.9% (P=0.000) (Figure 3).

### Prognostic analysis and Stage II group

Impact of prognostic factors on OS, DMFS and RC were evaluated using univariate and multivariate analyses, including age, gender, KPS, T stage, N stage, pretreatment PET/CT and the absence of chemotherapy (Table 2 and 3). Multivariate analyses indicated that N stage appeared to be prognostic factors for OS (P=0.000), RC (P=0.005) and DMFS (P=0.001). Age (P=0.004) and KPS (P=0.008) were independent predictors of OS and DMFS.

A total of 117 patients with stage II NPC were included in the study: 81 patients with chemo-radiotherapy and 36 patients with IMRT alone. OS (P=0.280), DMFS (P=0.245) and RC (P=0.505) were not significantly different in the absence of chemotherapy. Stage II patients with chemotherapy showed improved 5‐year and 10-year RC rate compared with RT alone patients (97.5% vs. 94.4% and 97.5% vs. 81.6%, P=0.013).

### Late toxicities

Overall, most late toxicities were assessed as grades 0 to 2. Seven patients had asymptomatic temporal necrosis detected by MRI scans, one of whom was recurrence and re-radiotherapy. Seven patients had cranial nerve palsy, and the possibility of recurrent disease was excluded by a series of MRI scans and physical examination. Single nerve palsy developed in six patients, including four patients with palsy of the hypoglossal nerve and one patient with recurrent laryngeal palsy, abducens nerve, respectively. One patient had two nerve palsies. One case of cranial nerve palsy occurred in the recurrence and after re-radiotherapy. Two patients were pathologically confirmed with osteonecrosis of mandible 8 years after the end of RT and 4 years after the end of re-radiotherapy. Eighteen patients had the second primary tumor, of whom eight were lung cancer, six were head and neck cancer, four were others (Table 4).

## Discussion

In this study, we retrospectively evaluated long-term outcomes and late toxicities of 276 T1-2N0-3M0 NPC, stage based on MRI and treatment with IMRT. We have published local control and acute toxicity in patients with T1-2 NPC. 13 This study was emphasis on long-term efficacy and late toxicity with more cases.

The dose of the target is critical to local control. With the advantages of conformal dose distribution and normal tissue protection, IMRT has replaced 2D-RT as the standard RT treatment for NPC 14-16. Zhang MX 17 evaluated the survival benefit of IMRT compared with 2D-CRT in 7081 non-metastatic NPC patients. At the time of 5 years, the patients administered IMRT had significantly higher loco-regional relapse-free survival (LRRFS) than those with 2D-CRT (92.5% and 88.5%, respectively). Subgroup analysis showed the LRRFS was higher for IMRT than 2D-CRT, with borderline significance in T1 Stage (97.6% and 93.3%, respectively; P=0.045) and significant difference in T2 Stage (95.8% and 90.2%, respectively; P=0.001). With the improved locoregionally control, distant metastasis changed into the main failure in advanced NPC after IMRT 17-21. The 5-year DMFS was 81.8-87.6% 17,19,21 and the 10-year was 79.8-83.4% 9,19-20. Wu LR reviewed the 10-year survival outcomes for patients with NPC receiving IMRT. 19 The 5-year and 10-year LRFS for T1 and T2 were 98.0% and 94.2%, 95.1% and 92.5%, respectively. The 5-year and 10-year DMFS were 81.8% and 79.8%. In our research, the 5-year and 10-year LC rate and DMFS were 97.0% and 91.9%, 89.4% and 87.0%, which were higher or similar to the previous study.

N-stages were part of different clinical stages which were known to be of prognostic value especially as a poor predictor of DMFS. The DMFS for N0-1 was significantly higher than that of N2-3 disease. 22-23 Yao et al. 22 compared clinical features and survival outcomes in patients with ascending type (type A:T3-4N0-1) and descending type (type D:T1-2N2-3) NPC in the IMRT era. Type D had a more aggressive clinical course of distant metastasis, regional recurrence, disease recurrence, and death (P < 0.001 for all) than type A. A retrospective study 23 from Sun Yat-Sen University Cancer Center analyzed 959 patients with N2-3 NPC. All patients received neoadjuvant chemotherapy of ≥3 cycles followed by IMRT. A propensity score matching was made between patients treated with/without concurrent chemotherapy (CCT). The 5-year OS, RFS (recurrence‐free survival) and DMFS for non-CCT and CCT were 77.7%, 87.4%, 78.9% and 73.4%, 86.0% , 73.8% (P=0.083, 0.225 and 0.248), respectively. Although neoadjuvant chemotherapy and CCT were added to IMRT, the distant metastasis was also the most important cause of death. In our study, most N1 stage (the diameter of lymph node ≥3 cm) and N2-3 stage patients received platinum‐based chemotherapy, patients with N0‐1 stage showed improved 5‐year and 10-year DMFS compared with N2-3 patients (97.9% vs. 80.1% and 95.7% vs. 77.5% (P=0.000)).

NPC patients in Stage II of different subgroups have heterogeneity and controversies exist around the management of chemotherapy. 24-28 Huang et al. 24 conducted a Phase 2 multicenter clinical trial in two groups (IMRT alone or CCRT) with stage II (2010 UICC/AJCC) NPC patients. They found that CCT added to IMRT did not improve survival or disease control. Survival outcomes of different groups with T1N1M0, T2N0M0 and T2N1M0 were not further analyzed considering the relatively small sample size. Compared with the results in Huang's research, another retrospective analysis 26 of stage II had different conclusions. 611 patients diagnosed with T1-2N0-1M0 NPC were included. The 5-year OS in the CCRT group was improved compared to RT only (80.5% vs 65.7%; P=0.0021). Multivariable analysis also showed improved survival with the addition of chemotherapy (Hazard ratio [HR] 0.59; 95 CI 0.39-0.89; P = 0.0124). Different subgroups had distinct survival outcomes in Stage II NPC with T1N1M0, T2N0M0 and T2N1M0 stage, respectively. This was a 10-year study, and treatment methods and technologies were constantly changing. The survival outcomes were not as satisfied as Huang's research. Guo et al. 27 found that T2N1 patients had significant poorer survival outcomes than T1N1 patients, with T2N0 patients in between. A total of 117 patients with stage II NPC were included in our study: 81 patients with chemo-radiotherapy and 36 patients with IMRT alone. OS (P=0.280) was not significantly different in the absence of chemotherapy. The role of concomitant chemotherapy cannot yet be exactly determined for the different subgroups of stage II.

IMRT was related to less acute and late toxicities compared with 2D-RT. The incidence, risk factors and degree of late toxicities were increased with more long-term survivors of NPC treated with definitive IMRT. 7, 9-10 Chen L et al. 9 verified 10-year results of survival and late toxicities and assessed the ultimate therapeutic ratio of IMRT versus 2DRT in patients with NPC. The incidence of grade 3-4 temporal lobe necrosis, cranial neuropathy, ear damage, neck soft tissue damage, trismus, and dry mouth was significantly lower in the IMRT group than the 2DRT group. In a retrospective study of 3328 NPC over a 10-year period, patients had late adverse reactions as follows: cranial nerve palsies (5.1%), hearing loss requiring hearing aids (7.1%), dysphagia requiring tube feeding for a long period (3%), and symptomatic temporal lobe necrosis (0.9%). 7 Wang L et al. 10 investigated long-term survivals and toxicities of 187 T1-2N0-1 NPC. With 15.7-year median follow-up, no grade 4 late toxicity happened; grade 3 late toxicities included subcutaneous fibrosis (4.3%), deafness or otitis (4.8%), skin dystrophy (2.1%) and xerostomia (1.1%). In our cohort, the incidence of 3-4 late toxicities were low and mainly xerostomia and hearing deficit. The rates of radiation-induced cranial nerve palsy and temporal necrosis were 2.5% and 2.5%, respectively.

Second primary tumor (SPT) is a serious late complication after IMRT for NPC. 29-30 The incidence and risk factors of SPT are poorly characterized. It can occur in-field or out-field anytime after RT and increases with long-term survivors of NPC. SPT is a dreadful complication and negatively impact of OS. It was observed that 290 cases SPT occurred with a crude incidence of 9.2% over a median follow-up period of 10.8 years after IMRT for NPC patients in Chow's study 29. It mainly included oral cavity, sarcoma, oropharynx, paranasal sinus, salivary gland, thyroid, skin and lung cancer. Additionally, Zhang et al 30 suggested 189 (3.0%) suffered SPT (median follow-up, 62 months) of the 6,377 patients. 14.3% suffered SPMs within 1 year post-IMRT: 1-3 years, 38.1%; 3-5 years, 33.9%; and >5 years, 13.7%. Lung cancer was the most common SPT (50/6,377, 0.78%). In NPC patients, the proportion of lung cancer after IMRT was similar to that of the normal population. Sex, age (≥50) and smoking history were significant risk factors for SPT, and the 5-year OS of NPC with/without SPT were 70.0% and 95.0%, respectively. Eighteen patients in our study had the second primary tumor, of whom eight were lung cancer, six were head and neck cancer, four were others.

## Conclusion

Satisfactory locoregional control was achieved in T1‐2 NPC treated with IMRT. Distant metastasis was the main failure cause and N2-3 was the main adverse prognostic factor, which was worthy of further study. Patients with long-term survival should pay more attention to the late toxicities including secondary tumors. Limitations of the study such as retrospective data and single center should be considered in the future research.

### Funding Sources

This work was supported by the Shanghai Anticancer Association EYAS PROJECT (grant no: SACA-CY20C06).

## Figures and Tables

**Table 1:** Characteristics of patients

Characteristic	No. of patients	Percent (%)
Age (years)		
Median 49 Range 17-78		
≤50	151	54.7
>50	125	45.3
Gender		
Male	204	73.9
Female	72	26.1
Histology		
Non‐keratinizing	271	98.2
Others	5	1.8
KPS Score		
90-100	147	53.3
70-80	129	46.7
T Stage		
T1	96	34.8
T2	180	65.2
N Stage		
N0	44	15.9
N1	99	35.9
N2	65	23.6
N3	68	24.6
Total Stage		
I	27	9.8
II	117	42.4
III	64	23.2
IV	68	24.6
PET-CT		
No	223	80.8
Yes	53	19.2
Chemotherapy (IC ± CCRT or AC)		
No	65	23.6
Yes	211	76.4

**Table 2:** Univariate analysis of prognostic factors

Characteristic	OS		RC		DMFS	
	P value	HR (95%CI)	P value	HR (95%CI)	P value	HR (95%CI)
Age (≤50/>50)	0.009	2.036 (1.193-3.473)	0.377	1.472 (0.625-3.471)	0.974	0.989 (0.502-1.946)
Gender (Male/Female)	0.897	0.960 (0.523-1.765)	0.442	1.428 (0.575-3.544)	0.115	0.466 (0.180-1.204)
KPS (90/100 and 70/80)	0.104	1.548 (0.914-2.623)	0.133	1.967 (0.814-4.753)	0.004	2.942 (1.406-6.157)
T stage (T1/T2)	0.171	1.500 (0.839-2.681)	0.045	3.488 (1.027-11.848)	0.417	1.358 (0.649-2.840)
N stage (N0-1/N2-3)	0.000	3.177 (1.778-5.675)	0.000	24.826 (3.330-185.092)	0.000	7.170 (2.774-18.532)
PET-CT (no/yes)	0.739	1.119 (0.578-2.166)	0.533	1.376 (0.504-3.756)	0.866	0.927 (0.384-2.237)
Chemotherapy (IC ± CCRT or AC) (no/yes)	0.366	1.356 (0.701-2.625)	0.067	6.525 (0.876-48.625)	0.023	5.225 (1.252-21.804)

**Table 3:** Independent prognostic factors by multivariate analyses

Endpoint	Factor	P value	HR(95%CI)
OS	Age (≤50/>50)	0.004	2.263 (1.306-3.920)
	KPS (90/100 and 70/80)	0.469	1.221 (0.712-2.095)
	T stage (T1/T2)	0.519	1.215 (0.672-2.195)
	N stage (N0-1/N2-3)	0.000	3.364 (1.862-6.078)
RC	KPS (90/100 and 70/80)	0.288	1.619 (0.666-3.938)
	T stage (T1/T2)	0.207	2.215 (0.645-7.606)
	N stage (N0-1/N2-3)	0.005	25.940 (2.676-251.454)
	Chemotherapy (IC ± CCRT or AC) (no/yes)	0.692	0.632 (0.065-6.109)
DMFS	Gender (Male/Female)	0.113	0.463 (0.179-1.198)
	KPS (90/100 and 70/80)	0.008	2.744 (1.306-5.765)
	N stage (N0-1/N2-3)	0.001	6.170 (2.104-18.094)
	Chemotherapy (IC ± CCRT or AC) (no/yes)	0.822	1.206 (0.237-6.122)

**Table 4:** Late severe toxicities for patients

Late severe toxicities	Total	Grade 3-4	Percent (%)
Temporal necrosis	7		2.5
Cranial nerve palsy	7		2.5
Osteonecrosis of Mandible	2		0.7
Fatal nasopharyngeal hemorrhage	0		0
Hearing deficit		16	5.7
Xerostomia		8	2.9
Neck fibrosis		2	0.7
Trismus		2	0.7
